# Retrospective CBCT analysis of maxillary sinus pathology prevalence in the Saudi Arabian population

**DOI:** 10.1016/j.sdentj.2024.03.016

**Published:** 2024-03-20

**Authors:** Abdulrahman Altwaijri, Shaul Hameed Kolarkdoi, Khalid Zabin Alotaibi, Faraj Alotaiby, Faris Jaser Almutairi

**Affiliations:** a Department of Oral and Maxillofacial Surgery, College of Dentistry, Qassim University, Qassim, Saudi Arabia; b Department of Oral and Maxillofacial Diagnostic Sciences, College of Dentistry, Qassim University, Qassim, Saudi Arabia

**Keywords:** Maxillary sinus, Odontogenic infection, Prevalence, CBCT, Sinusitis

## Abstract

**Introduction:**

Maxillary sinus pathology, particularly its association with odontogenic infections, is a significant concern in dentistry. This retrospective study explored the prevalence of maxillary sinus pathologies and their association with odontogenic infections in Saudi Arabia.

**Methods:**

This retrospective study included 411 patients aged 20–80 years. Cone-beam computed tomography was performed to identify the presence, location, and characteristics of odontogenic infections and maxillary sinus pathologies. Patients’ age and sex were also recorded.

**Results:**

Sinusitis was prevalent in 50.6 % of the cases, with no significant sex or age differences in the pathology distribution. A notable association was found between odontogenic and sinus pathologies, particularly on the left side.

**Conclusion:**

A significant prevalence of sinus pathologies, particularly sinusitis, was observed, with an association between odontogenic and sinus pathologies, notably on the left side. While acknowledging the limitations of the study, these findings underscore the need for integrated dental and sinus health assessments.

## 1 Introduction

Maxillary sinus pathology and its relationship with odontogenic infections are topics of significant concern in dentistry (Hameed et al., 2021). The maxillary sinus is an air-filled cavity located within the maxillary bone of the human skull and is one of four paired paranasal sinuses along with the others being the frontal, ethmoid, and sphenoid sinuses (Alnafisah et al., 2020). Maxillary sinus pathologies encompass a range of conditions that affect the sinus, including odontogenic maxillary sinusitis, mucous retention cysts (MRC), retention cysts (RC), and antral pseudocysts, which may or may not be associated with dental and periodontal infections, thickening of the sinus membrane (TSM), and radiological opacities (Whyte and Boeddinghaus, 2019).

Odontogenic infections originating from tooth structures are associated with a high prevalence of maxillary sinus pathologies (Curi et al., 2020). A recent prevalence study indicated that approximately 20–25 % of the population experiences maxillary sinus issues during their lifetime, with odontogenic infections contributing to around 10–15 % of these cases (Wuokko-Landén et al., 2021). The prevalence of chronic sinusitis in Saudi Arabia is estimated to be 25 % of the total population (Alfallaj et al., 2023). The upper molars and certain premolars, primarily the upper second and first molars, are anatomically positioned close to the maxillary sinus floor, rendering them more susceptible to odontogenic issues, including periapical lesions and periodontal diseases, often affecting the furcation areas. These teeth are typically separated from the maxillary sinus by a dense cortical bone layer; however, in some cases, they are only separated by the mucoperiosteum, leading to potential anatomical changes in the sinus membrane and radiological findings such as OMS, MRCs, and RCs. Among these, the TSM is the most frequently observed alteration (Yoshimine et al., 2012). The exact origins of MRCs, RCs, and antral pseudocysts remain debatable, as they may or may not be associated with dental and periodontal infections. Although some consider the maxillary sinus as normal in the absence of TSM or with uniform thickening below 2 mm, a consensus on the pathological threshold is lacking.

Cone-beam computed tomography (CBCT) offers valuable three-dimensional imaging for preoperative assessments and sinus health evaluations with lower radiation exposure than medical CT scans (Weiss and Read-Fuller, 2019). This retrospective study aimed to enhance our understanding of the prevalence of maxillary sinus pathologies and their association with odontogenic infections. The insights gained from this research will not only contribute to the existing body of knowledge but also serve as a valuable resource for clinicians, ultimately aiding in more informed and effective clinical approaches to address maxillary sinus-related issues associated with odontogenic infections.

## 2 Methodology

### 2.1 Study design and setting

This retrospective descriptive study was conducted at the Department of Dentistry, XXX University, between 2022 and 2023, following approval from the Ethics Committee of XXX University.

### 2.2 Study population

The study population comprised 411 records of patients aged 20–80 years retrieved from a specialized radiology center. The patients visited the clinic between 2022 and 2023 for dental procedures, including those related to odontogenic infections and maxillary sinus assessments.

### 2.3 Sample size

To determine the required sample size, a formula was employed considering a confidence level (CI) of 95 %, a margin of error (E) of 5 %, and an expected proportion (P) of 50 %. This calculation yielded a sample size of 411.

### 2.4 Inclusion and exclusion Criteria

Participants who met the technical quality standards for the precise assessment of odontogenic infections and maxillary sinus pathologies were included. Inclusion criteria were age between 20 and 80 years, regardless of sex, with accessible CBCT scans showing both the right and left maxillary sinuses. The exclusion criteria included a lack of accessible CBCT scans, inadequate scan quality, and incomplete demographic information. Patients with a history of chronic rhinosinusitis, allergic rhinitis, asthma, previous surgery, or facial malignancy were excluded. Additionally, images displaying alterations in the sinus or dental morphology due to trauma or pathological conditions were excluded.

### 2.5 Data collection

CBCT images were obtained using a CBCT unit (Sirona Dental Systems GmbH, Bensheim, Hessen, Germany; full FOV) under specified exposure conditions. Data collected during image analysis included the presence, location, and characteristics of odontogenic infections and maxillary sinus pathologies as well as patient’ age and sex. Image analysis was performed using a quantitative radiology software (Sidexis-XG software), and the data were recorded in Microsoft Excel. To ensure the reliability and consistency of the data, both inter- and intra-examiner agreements were assessed and maintained throughout the image analysis process using established protocols to minimize variability in the interpretation of CBCT images.

### 2.6 Statistical analysis

Descriptive statistics were used to summarize the data, including the prevalence, location, type, and characteristics of odontogenic infections and maxillary sinus pathologies. Chi-square tests were conducted to explore the potential associations between these findings and demographic factors, with the aim of identifying any significant relationships or differences. Statistical analyses were performed using IBM SPSS Statistics for Windows version 21.

## 3 Results

The findings of this study provide significant insights into the distribution of maxillary sinus pathologies in diverse patient populations. A range of sinus conditions were observed in the assessment of 411 participants, shedding light on the prevalence and diversity of these pathologies. Among the 411 participants, 238 were male (57.9 %) and 173 were female (42.1 %). Examination of odontogenic pathologies in the right (ODO-R) and left (ODO-L) maxillary sinuses concerning sex distribution did not yield statistically significant differences (p > 0.05). Table 1 illustrates the distribution of the maxillary sinus pathologies identified in the study. The most prevalent pathology was “Sinusitis,” observed in 50.6 % of cases, followed by “Normal” findings in 48.2 %. “Mucosal Thickening” was detected in 32.4 % of cases, while “ Pseudocyst” were identified in 9.5 % of participants. Other pathologies such as “Sinus Cysts,” “Sinus Polyps,” and “Sinus Malignancies,” were less frequent, with proportions ranging from 0.2 % to 2.4 %.

**Table 1:** Distribution of maxillary sinus pathologies.

Pathology	Right side (N)	Right side (%)	Left side (N)	Left side (%)
Normal	208	50.6	198	48.2
Maxillary aplasia	1	0.2	1	0.2
Pseudocyst	17	4.1	25	6.1
Mucosal Thickening	114	27.7	133	32.4
Sinusitis	46	11.2	39	9.5
Sinus cysts	10	2.4	7	1.7
Sinus polyps	10	2.4	5	1.2
Sinus malignancies	4	1.0	2	0.5
Multiple	1	0.2	1	0.2
Total	411	100.0	411	100.0

**Fig. 1:**
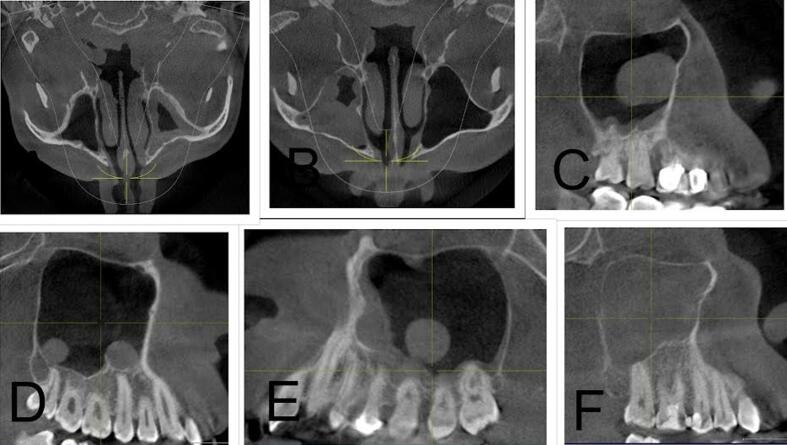
(A and B) Mucosal thickening, (C, D, and E) mucous retention pseudocyst, and (F) complete opacification without odontogenic infection.

**Fig. 2:**
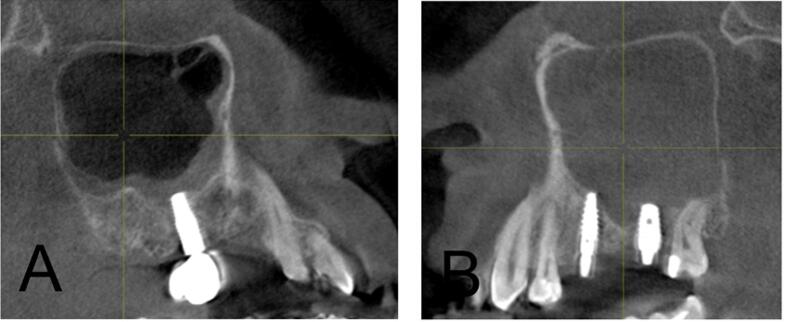
(A) Implant-associated mucosal thickening and (B) implant-associated complete opacification.

**Fig. 3:**
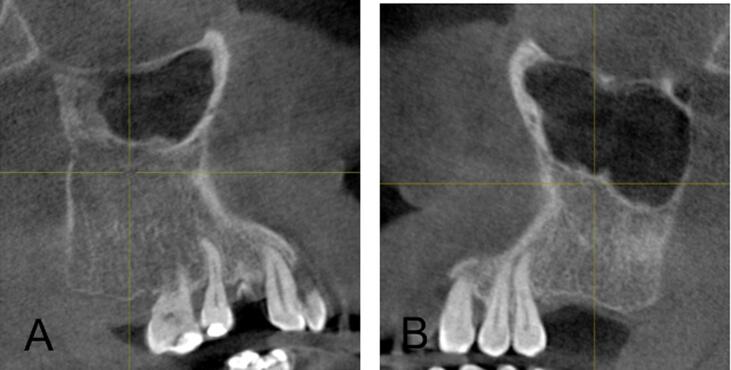
(A) Hypoplasia of the right maxillary sinus and (B) hypoplasia of the left maxillary sinus.

**Fig. 4:**
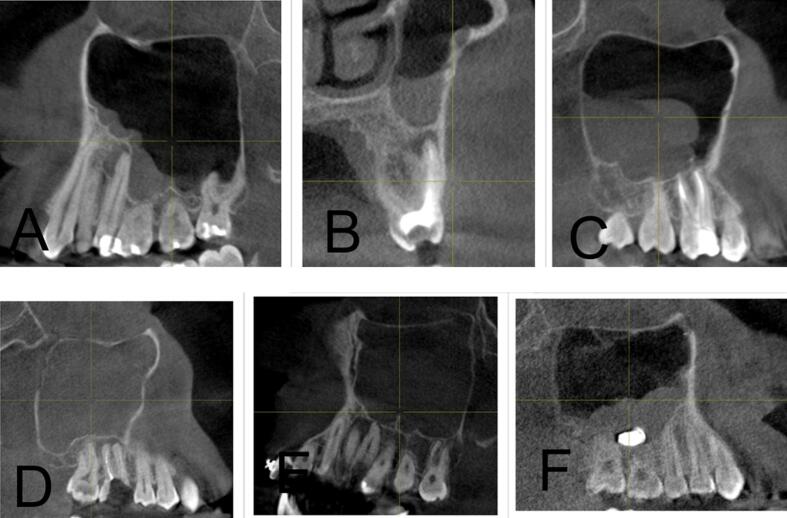
Various odontogenic-related maxillary sinus pathologies (A and B), mucosal thickening (C), polyp growth (D, E, and F), and complete opacification and mucosal thickening.

Table 2 summarizes the distribution of odontogenic pathology in the right (ODO-R) and left (ODO-L) maxillary sinuses within the study population. Periapical pathology was observed in 22.4 % on the right side and 28.7 % on the left side. Pathologies related to “Implant” were less common, comprising 1.9 % in ODO-R and 0.7 % in ODO-L, while “Others, i.e., odontogenic cyst” constituted a minimal proportion of 0.2 % in both ODO-R and ODO-L. Fig. 1 illustrates the various presentations of mucosal thickening in the examined cases where A and B depict instances of mucosal thickening; C shows polypoidal mucosal thickening; D and E highlight cases characterized by both mucosal thickening and sinus polyps; and F demonstrates complete opacification without evidence of odontogenic infection. Fig. 2 shows the implant-induced changes, revealing distinctive patterns. Specifically, A illustrates instances of mucosal thickening associated with implants, whereas B shows cases characterized by complete opacification attributed to implant-related factors. Fig. 3 shows cases of hypoplasia of the maxillary sinus with distinct observations on both sides where A illustrates hypoplasia on the right side, and B shows hypoplasia on the left side. Fig. 4 delineates various odontogenic-related maxillary sinus pathologies with a wide spectrum of manifestations wherein A and B depict instances of mucosal thickening associated with odontogenic factors, whereas C highlights polypoidal growth in the maxillary sinus. Additionally, D and E illustrate cases characterized by complete opacification with distinct mucosal thickening.

**Table 2:** Distribution of maxillary odontogenic pathologies for right and left maxillary sinuses.

Odontogenic pathology	Right side (N)	Right side (%)	Left side (N)	Left side (%)
No periapical pathology	310	75.4	289	70.3
Periapical pathology	92	22.4	118	28.7
Implant with pathology	8	1.9	3	0.7
Others	1	0.2	1	0.2
Total	411	100.0	411	100.0

A chi-square test was conducted to assess the relationship between Path-L (pathology in the left maxillary sinus) and ODO-L (odontogenic pathology in the left maxillary sinus) and Path-R (pathology in the right maxillary sinus) and ODO-R (odontogenic pathology in the right maxillary sinus). The results revealed a statistically significant association between these variables (chi-square = 279.656, df = 24, p < 0.001). This finding underscores the importance of considering both odontogenic and maxillary sinus pathologies when diagnosing and managing patients as they may be interconnected. There was no significant relationship between the odontogenic and maxillary sinus pathologies on the right side (p = 0.987).

## 4 Discussion

This study provides significant insights into the prevalence and characteristics of maxillary sinus pathologies and their association with odontogenic infections in the Qassim population. The study demonstrated a significant incidence of sinus pathologies, with sinusitis being particularly prevalent in 50.6 % of the cases. This rate correlates with findings from other studies. Kuligowski et al. reported a prevalence of up to 40 % for odontogenic sinusitis, while Al-Ehmeli et al., found a 37.21 % overall prevalence of sinus pathology in their patient cohort (Kuligowski et al., 2021, Al-Ehmeli, 2015). In contrast, Mahasneh et al. observed a notably higher prevalence, with 76.1 % of patients showing incidental pathological findings (Mahasneh et al,. 2022). These results highlight the global relevance of sinus pathologies in dental practice, emphasizing a common challenge encountered by dental professionals worldwide. Sinusitis can often mimic toothache symptoms, adding complexity to the diagnosis and treatment of maxillary sinus pathologies in dental settings.

In the realm of sinus pathologies, a professional distinction is made between odontogenic and non-odontogenic categories. Odontogenic pathologies generally originate from dental conditions, such as infections spreading from the teeth to the sinuses (Psillas et al., 2021). Non-odontogenic sinus pathologies are divided into two subcategories: inflammatory and non-inflammatory (Zhang et al., 2023). Inflammatory pathologies encompass conditions like chronic rhinosinusitis with nasal polyps, chronic rhinosinusitis without nasal polyps, allergic fungal sinusitis, and fungal balls. These are often precipitated by systemic factors, such as infections or allergies. The noninflammatory category includes conditions such as inclusion cysts, choanal polyps, and rare malignancies that are typically unrelated to dental health. This delineation is critical for precise diagnosis and appropriate management of sinus conditions (Fischborn et al., 2023).

The current study, along with studies by Nunes et al., Shanbhag et al., Maillet et al., Liu et al., and Nascimento et al., consistently revealed a significant link between odontogenic pathology and maxillary sinus abnormalities, particularly on the left side (de Souza-Nunes et al., 2019, Shanbhag et al., 2013, Maillet et al., 20111, Lu et al., 2012, Nascimento et al., 2016). These findings collectively highlight the substantial influence of dental health on sinus conditions, with Nascimento et al. (2016) suggesting dental causes in more than half of maxillary sinusitis cases. This convergence of evidence emphasizes the need to consider dental factors when assessing and treating sinus pathologies. However, studies by Siviero et al., Phothikhun et al., Goller-Bulut D, and Eduarda et al. presented contrasting findings, revealing a lack of association between odontogenic diseases and certain maxillary sinus pathologies, including mucosal changes and sinusitis (Siviero et al., 2020, Phothikhun et al., 2012, Goller-Bulut et al., 2015, Pelinsari Lana et al., 2012). This indicates that factors beyond odontogenic sources may be more significant in the development of specific sinus pathologies.

Kumar et al. emphasized the distinct condition of OMS recognized by both dental and otolaryngology communities (Nandakumar et al., 2021). Accounting for approximately 30 % of unilateral maxillary sinusitis cases, OMS has a substantial association with odontogenic sources. Shaoqing et al. reported varied findings, with some studies identifying odontogenic infection as a predisposing factor for pathological disorders in the maxillary sinus, hinting at a potential link between dental health and sinus conditions (Shaoqing et al., 2021). These varied outcomes underscore the intricate nature of the relationship between odontogenic factors and maxillary sinus pathologies, emphasizing the importance of considering multiple factors in patient evaluation of potential odontogenic infections. This study also draws attention to the distinct prevalence of primary odontogenic factors, such as periodontal issues, apical lesions, and dental cysts, as well as secondary or iatrogenic factors, such as *peri*-implantitis, oroantral fistula, and sinus floor elevation complications, in our region. Differentiation between these factors is crucial for understanding the nuanced interplay between dental health and sinus pathologies in specific demographics.

The present study did not find significant sex or age differences in the distribution of odontogenic pathologies, a point that diverges from other studies suggesting slight sex-based variations. This discrepancy can be attributed to demographic differences. This study also acknowledges the limitations of fully elucidating the reasons for the observed left-sided prevalence of sinus pathologies. Further research is required to explore the underlying factors specific to our study population that may contribute to this asymmetry. Despite these limitations, this study highlights the importance of integrated dental and sinus health assessments. To enhance the reliability of these insights, future research should consider employing diverse methodologies and larger, more representative sample sizes.

## 5 Conclusion

The research revealed a substantial prevalence of sinus pathologies, particularly sinusitis, affecting 50.6 % of the cases. Notably, no significant variations were observed in sex or age with respect to the distribution of sinus or odontogenic pathologies. However, a noteworthy association emerged between odontogenic and sinus pathologies, particularly on the left side, among the study participants.

## Declaration of competing interest

The authors declare that they have no known competing financial interests or personal relationships that could have appeared to influence the work reported in this paper.
